# Prevalence of Postpartum Depression Based on Diagnostic Interviews: A Systematic Review and Meta-Analysis

**DOI:** 10.1155/2023/8403222

**Published:** 2023-08-19

**Authors:** Yanping Bai, Qiao Li, Kar Keung Cheng, Eric D. Caine, Yongsheng Tong, Xia Wu, Wenjie Gong

**Affiliations:** ^1^HER Team and Department of Maternal and Child Health, Xiangya School of Public Health, Central South University, Hunan 410078, China; ^2^Institute of Applied Health Research, University of Birmingham, B15 2TT Birmingham, UK; ^3^Department of Psychiatry, University of Rochester, Rochester, New York, USA; ^4^Beijing Suicide Research and Prevention Center, Beijing Hui Long Guan Hospital, 7 Nan Dian Road, Changping, Beijing 100096, China

## Abstract

**Background:**

Postpartum depression (PPD) is common after childbirth. Previous reviews on the prevalence of PPD have mainly included results that relied on screening instruments or a mixture of such instruments and diagnostic interviews. In this study, we aimed to assess the prevalence of PPD based exclusively on studies using diagnostic interviews, as they provide the most reliable and valid approach for defining “caseness.”

**Methods:**

Using PubMed, Web of Science, Cochrane Library, Embase, CNKI, WANFANG DATA, and CBM up to September 18, 2022, we searched for original articles reporting data that could be used to calculate the prevalence of PPD based on diagnostic interviews. A random-effect meta-analysis model was then used to estimate the pooled prevalence. In addition, we assessed quality, heterogeneity, and publication bias across studies. Also, we did subgroup analyses to explore the pooled prevalence at different time points and settings. This study was registered with PROSPERO, CRD42021244539.

**Results:**

Of 17,115 articles retrieved, 54 studies were included (total sample size = 15,586 women). The pooled prevalence of all depression and major depression within one year postpartum was 12.1% (95% CI 10.3%-14.1%; *I*^2^ = 91.0%) and 7.0% (95% CI 5.7%-8.4%; *I*^2^ = 83.0%), respectively. The peaks of all depression occurred during the first 6 months postpartum, especially 2-3 weeks and 6-8 weeks. Subgroup analyses showed that the prevalence of major depression was associated with the income level of countries (higher in low- and middle-income countries (LMICs) than in high-income countries (HICs)) and diagnostic criteria (higher using ICD than using DSM and RDC). No evidence of publication bias was found.

**Conclusions:**

Approximately one in eight postpartum women experiences a depressive condition, with one in fifteen suffering major depression. The pooled prevalence based on diagnostic interviews was lower than the existing consensus, which was largely based on self-reported screening instruments. The higher prevalence in LMICs underlines the importance of strengthening research and service provision among these populations.

## 1. Introduction

Postpartum depression (PPD) is a depressive episode that occurs following childbirth, characterized by a persistent depressed mood and a loss of interest or pleasure in most activities [1, 2]. The time frames for diagnosing PPD vary with different classification systems. The Diagnostic and Statistical Manual of Mental Disorders, fifth edition (DSM-5), defines it as occurring within 4 weeks postpartum [1], and the International Statistical Classification of Diseases, tenth revision (ICD-10), sets the temporal definitions within 6 weeks postpartum [2], while World Health Organisation (WHO) and the Centers for Disease Control and Prevention (CDC) extend the risk period to 12 months postpartum [3, 4]. Making a firm diagnosis of PPD requires a diagnostic interview. However, of the 33 relevant systematic reviews on the prevalence of PPD we identified, eleven had included studies that only used results from self-report screening instruments (prevalence of PPD ranging from 10.7% to 27.0%) [5–15]. The other 21 reviews included a mixture of studies using screening instruments only or screening followed by diagnostic interviews (prevalence ranging from 13.0% to 30.0%) [16–36]. Of these, two explicitly recognized the conceptual difference between screening and diagnostic interviews and reported prevalence estimates of studies stratified accordingly. However, one of them included only Asian women [23], and the other excluded women with a history of depression [28]. We found one systematic review that only included studies that used diagnostic interviews after screening [37]. However, this review focused on economically developed countries and included studies published only to 2004. It found that the prevalence of depressive episodes and major depressive episodes was 19.2% and 7.1%, respectively, during the first 3 months postpartum period. In addition, while there are papers synthesizing the prevalence of perinatal depression through individual patient data meta-analyses (IPDMA), they did not provide subgroup analysis with a distinction between pregnancy and postpartum [38–41].

Of particular importance, estimates of the prevalence of PPD reported in published reviews often were cited in authoritative documents without noting these substantive limitations. For example, the main supporting reference cited in a U.S. Department of Health & Human Services [42] was a systematic review in which only five of the 53 reviewed studies used diagnostic interviews when producing their prevalence estimates [17]. Also, the section on PPD in a recent report of the *Lancet Commission on 70* years *of Women's Reproductive, Maternal, Newborn, Child, and Adolescent Health in China* [43] cited only one systematic review [15] in which none of the seventeen papers included was based on diagnostic interviews. Given that self-report screening instruments are not diagnostic and cannot be used confidently alone to establish “caseness” of a clinical condition without qualification [44], this practice calls into question whether accurate estimates of the prevalence of PPD have been considered when formulating important guidelines and policies.

In this paper, we aimed to conduct a systematic review on the prevalence of PPD based solely on studies that included diagnostic interviews. Furthermore, we also conducted subgroup analyses according to study characteristics, including income level of countries/regions, time point postpartum, and diagnostic interview methods.

## 2. Methods

The proposal for this systematic review has been registered with PROSPERO under registration number: CRD42021244539. The manuscript follows the preferred reporting items for systematic reviews and meta-analysis (PRISMA) statement guidelines in the reporting process.

### 2.1. Search Details

We searched in four English databases (PubMed, Web of Science, Cochrane Library, and Embase) and three Chinese databases (CNKI, WANFANG DATA, and CBM) combining the following MeSH terms and free terms: (1) postpartum⁣^∗^, puerper⁣^∗^, postnatal⁣^∗^, perinatal⁣^∗^, matern⁣^∗^; (2) depress⁣^∗^, mental disorder⁣^∗^, dysthymi⁣^∗^, affective⁣^∗^, mood; (3) prevalen⁣^∗^, inciden⁣^∗^, frequen⁣^∗^, rate⁣^∗^, occurr⁣^∗^, epidemi⁣^∗^, etc. from the time the database was created until September 18, 2022. References of the included studies that met the inclusion criteria were also included in the analysis as supplementary ones.

### 2.2. Selection Criteria

*Inclusion criteria:* (1) cohort studies or cross-sectional studies; (2) used diagnostic interviews to assess PPD and reported data that could be used to calculate the prevalence of PPD based on diagnostic interviews; (3) original studies published in peer-reviewed journals. If the original publication examined perinatal depression—both preterm and postpartum—only those that reported the prevalence of PPD, or contained data that could be used to calculate prevalence, were included. We had no restrictions on the diagnostic interview protocol, including both structured and semistructured interviews, and we had no restrictions on diagnostic criteria (e.g., DSM, ICD, and RDC).

*Exclusion criteria:* (1) original studies did not conduct diagnostic interviews for all included mothers; (2) studies with mothers younger than 18 years of age; (3) studies whose reports were published in languages other than English or Chinese; (4) studies with special groups including those who were HIV-infected or AIDS, refugees, migrants, children born prematurely, and children in ICU.

### 2.3. Data Extraction

Two researchers independently screened the eligible primary studies and then performed data extraction and referred to a third researcher for judgment in case of dispute. Extracted data included information such as country/region, year of publication, type of study design, sample source, and sample size; information related to the diagnostic interviews included criteria for diagnosis (ICD/DSM/RDC, etc.), specific diagnostic interviews protocols (e.g., Structured Clinical Interview for DSM (SCID)/Mini International Neuropsychiatric Interview (MINI)), specific time point of the interview (e.g., six weeks postpartum), interview diagnostic outcome classification, number of patients in each category, mode of interviews (e.g., face-to-face and telephone), and professional background of interviewers (mental health professional and lay interviewers). MINI, Composite International Diagnostic Interview (CIDI), the Clinical Interview Schedule (CIS), and Diagnostic Inventory Schedule (DIS) were classified as structured interviews; SCID, Schedule for Affective Disorders and Schizophrenia (SADS), Present State Examination (PSE), the Birmingham Interview for Maternal Mental Health (BIMMH), and Diagnostic Interview for Genetic Studies (DIGS) were classified as semistructured interviews. We first recorded the diagnosis according to the definition and name of the diagnostic classification used in the original studies (e.g., DSM, ICD, and RDC) and then summarized and processed them during data analysis to deal with variations in classification. DSM-IV, for example, classifies depression into minor depression and major depression, as well as “other” depressive disorder and “unspecified” depressive disorder for syndromes that do not conform to a sufficient number or specific criteria. Depressive conditions can be subdivided into mild/moderate/moderate-severe/severe depending on the number of symptoms, although many publications based on RDC only report case/noncase. If the original paper reported data from multiple time points, data from each were included and extracted for later selected analyses.

### 2.4. Quality Assessment

We used the Loney criteria to assess the quality of observational studies [45]. The score range of this scale is 0-8. We assigned scores of 0-3, 4-6, and 7-8 for low-, moderate-, and high-quality studies, respectively.

### 2.5. Data Analysis

We first grouped diagnostic results into an “all depression” cluster inclusive of “depression,” “minor depression,” “major depression,” “major depressive episode/disorder,” “mild/moderate/severe/moderate-severe depression,” and “depression not otherwise specified.” The included diagnosis of the original studies was further categorized as “major depression” if it was reported as “major depression,” “major depression episode/disorder,” and “mild/moderate/severe/moderate-severe depression.” When the diagnosis reported in the original study was classified as “case/noncase” or “patient/nonpatient” or “depressed/nondepressed,” making it impossible to determine whether it was “major depression,” the original study was included in the analysis only when “alldepression” was calculated.

Time point prevalence is the proportion of a population that has the characteristic at a specific point in time, such as the four weeks postpartum. For cohort studies which reported data from multiple time points, we included data from each time point as reported if it was used to assess the prevalence of PPD at different time points. If it was only used to calculate the pooled prevalence of “all depression” or “major depression” or other subgroup analysis such as in high-income countries/regions (HICs) vs. low- and middle-income countries/regions (LMICs) [46], only data from the first time point postpartum in the study would be used.

Meta-analysis was carried out using “Meta” packages [47] implemented in R Statistical Software with random-effects meta-analysis model (to account for heterogeneity following assessment of the *I*^2^ statistic, *I*^2^ > 50% was regarded as showing significant heterogeneity) and double arcsine transformation. Pooled prevalence estimates were reported using 95% confidence interval (CI) with the results and 95% CI back transformed for ease of interpretation. We split the included studies by subgroup for analysis to explore prevalence at different time points and settings. We tested for the possibility of publication bias using “funnel plot.”

## 3. Results

**Figure 1:**
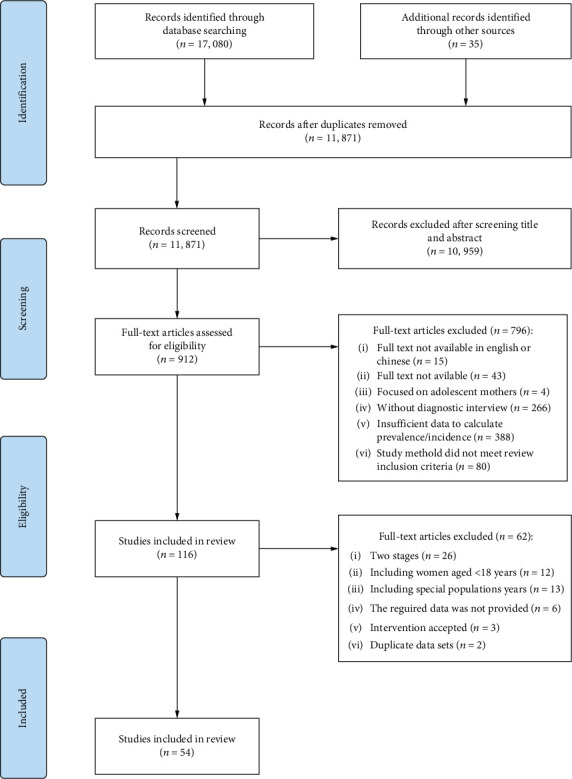
Flow chart of studies included in the systematic review.

An initial search yielded a total of 17,115 articles. After excluding duplication, 11,871 articles were obtained, and 912 original studies were initially included after screening by inspecting the titles and abstracts. After assessing the full text of these articles, 54 were finally included. See Figure 1 for details.

### 3.1. Prevalence of Postpartum Depression

A total of 54 studies from 1982 to 2022 reported all depression prevalence, with a total sample of 15,586. Sizes of samples ranged from 45 to 3,015. Thirty-three reports came from HICs. These included the United Kingdom (*n* = 9), the United States (*n* = 5), Australia (*n* = 3), Portugal (*n* = 2), and one each from Canada, Chile, China (Hong Kong SAR), France, Germany, Greece, Hungary, Italy, Japan, Malta, Singapore, Switzerland, and the United Arab Emirates. One report was based on 8 HICs (Australia, France, Iceland, Italy, Portugal, Switzerland, the United Kingdom, and the United States). Twenty-one reports were based on LMICs. These included Brazil (*n* = 4), China (*n* = 4), India (*n* = 2), Nigeria (*n* = 2), Turkey (*n* = 2), Vietnam (*n* = 2), and one each from Eritrea, Mexico, Morocco, Nepal, and Uganda. Twenty-seven were prospective studies, and 27 were cross-sectional studies. The basic characteristics of the studies are displayed in STable 1.

**Figure 2:**
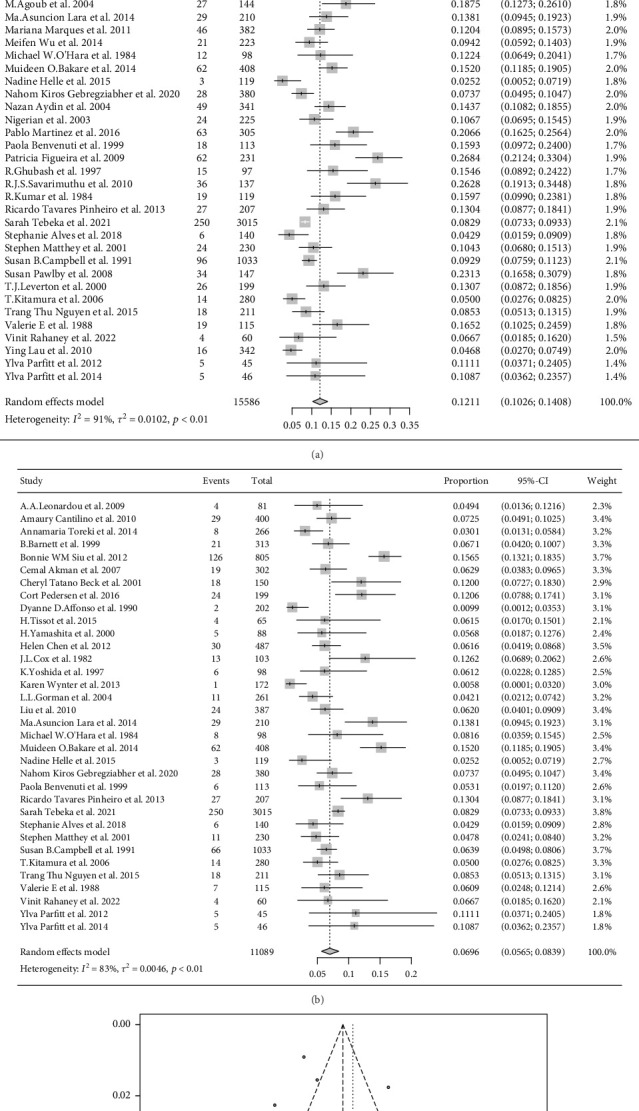
(a) Forest plot of the prevalence for all depression. (b) Forest plot of the prevalence for major depression. (c) The funnel diagram of the prevalence for all depression.

The pooled prevalence of all depression was 12.1% (95% CI 10.3%-14.1%; *I*^2^ = 91.0%), and the pooled prevalence of major depression was 7.0% (95% CI 5.7%-8.4%; *I*^2^ = 83.0%). See Figures 2(a) and 2(b) for a detailed forest plot.

### 3.2. Prevalence of Different Postpartum Time Points

**Figure 3:**
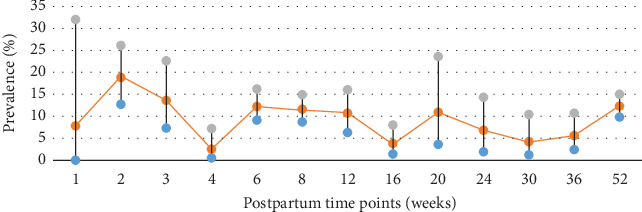
Point prevalence of all depression. The pooled prevalence and 95% CI at each time point were shown.

Thirty-four of the 54 studies reported time point prevalence, with a total of thirteen time points reported, ranging from one week to one year postpartum. The more frequently reported time points were 6 weeks postpartum (*n* = 13), 8 weeks postpartum (*n* = 6), 12 weeks postpartum (*n* = 8), and 6 months postpartum (*n* = 4). The pooled prevalence of all depression was higher during the first 6 months postpartum, especially at 2-3 weeks and 6-8 weeks postpartum. See Table 1 and Figure 3 for more details.

### 3.3. Prevalence of Subgroup Analysis

We conducted subgroup analyses of prevalence under different conditions. The results showed that the pooled prevalence of major depression was higher in studies in LMICs than in HICs, and that the pooled prevalence of major depression based on ICD diagnostic criteria was higher than in those using DSM and RDC. The differences were statistically significant (*P* < 0.05). There were no statistically significant differences in the pooled prevalence of all depression and major depression in the other subgroups, including setting for the interview, professional background of interviewers, interview protocol, and mode of interviews (*P* > 0.05). Details are presented in Table 2.

We assessed the pooled prevalence of all depression in individual countries/regions with a high number of studies. These included the United Kingdom (*n* = 9, pooled prevalence 15.3% (95% CI 12.9%-18.0%)), the United States (*n* = 5, pooled prevalence 11.5% (95% CI 4.8%-20.4%)), Brazil (*n* = 4, pooled prevalence 13.3% (95% CI 5.8%-23.2%)), China (*n* = 4, pooled prevalence 15.3% (95% CI 5.5%-28.8%)), and Australia (*n* = 3, pooled prevalence 5.0% (95% CI 0.9%-12.2%)). And the pooled prevalence of major depression in the United States (*n* = 5) and Australia (*n* = 3) was 7.1% (95% CI 3.4%-11.8%) and 3.6% (95% CI 0.8%-8.2%), respectively.

### 3.4. Sensitivity Analysis and Quality Evaluation

**Figure 4:**
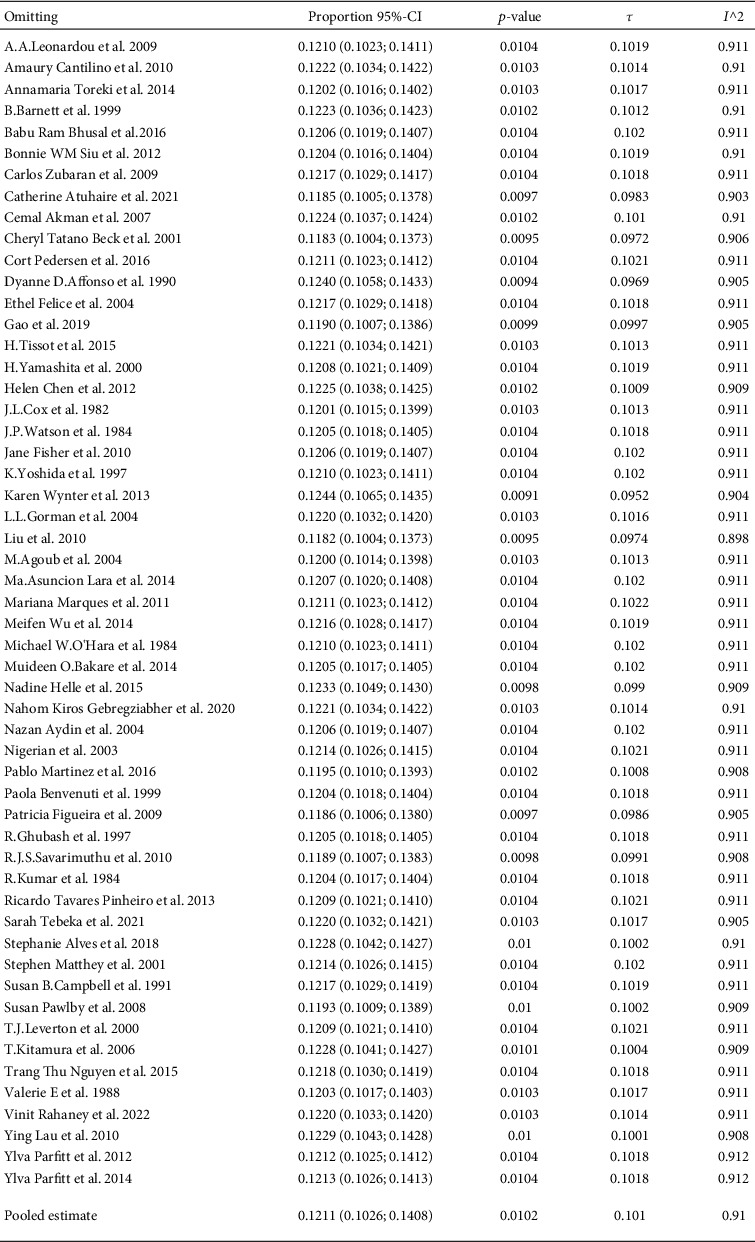
Sensitivity analysis results.

Figure 4 displays the sensitivity analysis results. The heterogeneity was found to be about 91% after the exclusion of one study at a time. After excluding the two studies with sample sizes less than 50, a heterogeneous result of 91.3% (95% CI 89.4%-92.9%) was still observed. This indicates a significant heterogeneity between studies.

The quality assessment scores of included studies are displayed in STable 2. Twenty-five scored 0-3 (low-quality), 28 scored 4-6 (moderate-quality), and one scored 7 (high-quality). There was no significant difference in the pooled prevalence between studies deemed to have poorer quality and those with better study quality scores (*P* > 0.05). See Table 2 for more details.

### 3.5. Publication Bias

The funnel plot of all depression prevalence is shown in Figure 2(c). There was no obvious bias in the original studies included in this systematic review (*P* = 0.13).

## 4. Discussion

This systematic review and meta-analysis examine the prevalence of PPD based on diagnostic interviews in both HICs and LMICs. These results improve our understanding of the epidemiology of PPD and provide a more informed basis for estimating the burden of care and resource allocation for the improvement of maternal mental health. Including original studies from a total of 28 countries/regions in four continents, we found that approximately one in eight postpartum women suffered from a depressive condition within one year postpartum, and one in fifteen experienced major depression. These were substantially lower than previous estimates based on information arising from studies using only results from screening instruments (for example, 17.7% as reported in the largest previous review, which included 291 studies) [5].

A previous systematic review based on diagnostic interviews dealt solely with HICs [37]. In our review, the pooled prevalence of all depression and major depression within one year postpartum in LMICs was 13.9% and 9.2%, respectively—higher than those in HICs (10.9% and 6.2%, respectively). In view of the scarcity of resources and relatively higher number of pregnancies and births in LMICs [48], the higher prevalence of PPD would bring considerable challenges. As major depression is likely to have more significant health and social impacts, the priority in these countries should focus on the identification and management of women who suffer from this condition.

The peak prevalence of PPD is currently disputed [49, 50], which not only affects the definition of PPD but also leads to debate on the screening time points for PPD. The peak prevalence we found for all depression varied during the first year postpartum, with peaks concentrated in the first 6 months postpartum, especially at 2-3 weeks postpartum and 6-8 weeks postpartum. However, the two main current classification systems (DSM and ICD) do not extend the temporal definitions to 8 weeks for the definition of PPD. [1, 2] Given our finding, these may be a case for extending the time window for the onset criteria of PPD.

Our analysis found no statistically significant impact of the interview setting and interviewer's professional background on the prevalence rates. However, further exploration is needed to understand the factors influencing diagnostic accuracy before the broader implementation of these practices. In addition, previous reviews have shown that reported prevalence rates of major depression results based on semistructured interviews were higher than those from structured interviews [39, 51]. However, our review showed no difference between them. In general, lay interviewers used structured interviews that require less professional training, but six of ten studies in this review employed lay interviewers to carry out semistructured interviews.

We also found that studies using ICD as a diagnostic criterion reported a significantly higher prevalence of PPD than DSM and RDC, which is consistent with the results of Wittchen's study [52]. This may be related to the small number of ICD-based studies or the possibility of a lower threshold using ICD diagnostic criteria [53]. Finally, the findings of this review reveal a difference that just failed to reach statistical significance in the pooled prevalence rates of major depression between studies using telephone interviews and face-to-face interviews. The limited number of studies utilizing telephone interviews was likely to be a factor behind the lack of statistical significance. Further comparative research would be beneficial in gaining a comprehensive understanding of the variations in prevalence estimates between telephone and face-to-face interviews. With the increase in popularity of online interviews during the pandemic [54], this question warrants further studies. Meanwhile, the literature about telephone included in this manuscript did not use camera interview, so future research should pay attention to the different influences of telephone interviews with a camera on diagnosis.

This review has several limitations. We included only studies written in English and Chinese; due to resource and technical limitations, we did not have access to databases like PsychINFO, which may cause potential omissions, despite having also systematically reviewed references from the included literature and other systematic reviews. We did not specify the sample size of the studies as an inclusion criterion: two of the studies included had sample sizes less than 50 individuals. These small investigations were likely to have increased the heterogeneity among the studies. Despite their small sample size, our sensitivity analysis showed that these studies did not contribute significantly to the heterogeneity among the included studies. Also, nearly half of the studies were considered to be of low-quality though subgroup analyses did not reveal major differences in the prevalence of PPD obtained from low-quality compared to moderate- and high-quality studies. Furthermore, of the 54 original studies included, only 20 aimed at the studying of prevalence of PPD, while the remaining studies focused on scale validation and exploration of the risk factors for PPD or for other reasons. This should be borne in mind in interpreting the findings. Finally, we found high heterogeneity among studies, which may affect the stability and reliability of the findings. We suggest that future meta-analysis studies should consider more possible influencing factors in the design stage in order to reduce heterogeneity and improve the precision and reliability of the study results.

## 5. Conclusions

In summary, this systematic review and meta-analysis examine the prevalence of PPD based on diagnostic interviews in both HICs and LMICs. We found that the pooled prevalence of PPD based on diagnostic interviews was lower than previous estimates. This finding is important for the understanding of the epidemiology of this condition and may provide a better basis for planning the allocation of health resources. We found in LMICs a higher prevalence of PPD. Together with their higher birth rates, these results suggest that service provision and research need to be urgently strengthened in these countries. In addition, specific study characteristics have an impact on prevalence, such as the timing of interviews and diagnostic criteria. These may help inform the design of future research on PPD.

## Figures and Tables

**Table 1:** Prevalence of different postpartum time points.

Postnatal time points	All depression		Major depression	
	Number of studies	Prevalence(95% CI)	Number of studies	Prevalence(95% CI)
1 w	2	7.8% (0; 32.0%)	1	1.0% (0.1%; 3.5%)
2 w	1	18.8% (12.7%; 26.1%)	NA	NA
3 w	1	13.6% (7.3%; 22.6%)	1	5.7% (1.9%; 12.8%)
4 w	1	2.5% (0.5%; 7.2%)	1	2.5% (0.5%; 7.2%)
6 w	13	12.2% (9.1%; 16.2%)	7	6.4% (4.5%; 8.5%)
8 w	6	11.6.% (8.7%; 14.9%)	4	9.4% (5.1%; 14.6%)
12 w	8	10.7% (6.3%; 16.0%)	4	4.3% (0.0%; 13.7%)
16 w	1	3.8% (1.4%; 8.0%)	1	4.3% (1.6%; 9.1%)
20 w	1	10.9% (3.6%; 23.6%)	1	10.9% (3.2%; 23.6%)
24 w	4	6.8% (1.9%; 14.3%)	3	5.4% (0.5%; 14.4%)
30 w	1	4.2% (1.2%; 10.4%)	NA	NA
36 w	1	5.6% (2.4%; 10.7%)	NA	NA
52 w	3	12.3% (9.8%; 15.0%)	2	11.1% (7.4%; 15.6%)
Total	34	11.1% (9.1%; 13.3%)	20	6.3% (4.6%; 8.1%)

Note: NA: not applicable.

**Table 2:** Subgroup analysis of prevalence of postpartum depression (PPD).

Subgroup	All depression			Major depression		
	Number of studies	Pooled prevalence (95% CI)	*P* value	Number of studies	Pooled prevalence (95% CI)	*P* value
Quality evaluation						
Low-quality	25	12.2% (9.3%; 15.4%)	0.81	17	7.6% (5.1%; 10.5%)	0.41
Moderate- and high-quality	29	11.9% (9.7%; 14.3%)		17	6.6% (5.2%; 8.1%)	
Primary purpose of research						
To study prevalence of PPD	20	10.3% (7.5%; 13.5%)	0.14	14	6.9% (4.7%; 9.5%)	0.93
Others	34	13.2% (10.9%; 15.8%)		20	7.0% (5.2%; 9.0%)	
Type of study						
Cohort	27	11.0% (8.9%; 13.2%)	0.25	16	6.8% (4.4%; 9.6%)	0.82
Cross-sectional	27	13.2% (10.4%; 16.3%)		18	7.0% (5.4%; 8.8%)	
Country/region income level						
High-income	33	10.9% (9.0%; 13.0%)	0.15	25	6.2% (4.7%; 7.8%)	0.04
Low- and middle-income	21	13.9% (10.7%; .17.4%)		9	9.2% (6.9%; 11.7%)	
Recruiting site						0.98
Medical institution	46	12.1% (10.2%; 14.0%)	0.91	29	7.0% (5.7%; 8.4%)	
Nonmedical institution	8	12.4% (6.5%; 19.8%)		5	6.8% (2.3%; 13.4%)	
Setting for the interview						
Medical institution	24	12.6% (10.0%; 15.5%)	0.52	13	7.4% (5.0%; 10.1%)	0.87
Others	26	11.3% (9.1%; 13.6%)		18	7.0% (5.3%; 8.8%)	
Professional background of interviewers						
Mental Health professional	29	12.8% (10.6%; 15.2%)	0.28	18	7.8% (6.4%; 9.4%)	0.10
Lay interviewers	14	10.2% (6.6%; 14.4%)		10	5.3% (3.3%; 7.7%)	
Interview protocol						
Semistructured interview	37	11.6% (9.6%; 13.8%)	0.70	25	6.8% (5.4%; 8.3%)	0.77
Structured interview	14	12.6% (9.0%; 16.7%)		8	7.5% (4.3%; 11.4%)	
Diagnostic criteria						
DSM^a^	33	11.2% (8.9%; 13.7%)	0.24	23	6.6% (5.2%; 8.1%)	<0.01
ICD^b^	7	15.3% (11.6%; 19.4%)		1	15.2% (11.9%; 19.1%)	
RDC^c^	8	11.2% (7.2%; 15.9%)		6	5.1% (2.9%; 7.9%)	
Mode of interviews						
Face-to-face	44	12.3% (10.4%; 14.3%)	0.24	26	7.7% (6.1%; 9.4%)	0.08
Telephone	7	8.7% (5.3%; 12.8%)		6	4.9% (2.9%; 7.4%)	

Note: a: diagnostic and statistical manual of mental disorders; b: international classification of diseases; c: research diagnostic criteria.

## Data Availability

Study data are available on request to the corresponding author at gongwenjie@csu.edu.cn.

## Additional Points

*Key Messages*. *Question*. What is the prevalence of postpartum depression (PPD) based exclusively on studies using diagnostic interviews instead of relying on screening instruments? *Findings*. Approximately one in eight postpartum women experiences a depressive condition, with one in fifteen suffering major depression. The pooled prevalence based on diagnostic interviews was lower than the existing consensus, which was largely based on self-reported screening instruments*. Meaning*. Our findings provide more accurate bases for future studies of the epidemiology of PPD, clinical practice for women suffering from the condition, and relevant healthcare planning.

## Supplementary Materials

Supplementary Materials The supplementary file includes basic characteristics of all included studies (STable1), the results of their quality assessment (STable2), and references.
