# Supplementary material for: Prevalence of Postpartum Depression Based on Diagnostic Interviews: A Systematic Review and Meta-Analysis
Source: Depress Anxiety. 2023 Aug 19;2023:8403222. doi: 10.1155/2023/8403222 (PMC11921862; doi:10.1155/2023/8403222)
Supplement: Supplementary Materials — The supplementary file includes basic characteristics of all included studies (STable1), the results of their quality assessment (STable2), and references. [file 8403222.f1.docx]

**STable1：Basic Characteristics of Studies of the Prevalence of Postpartum depression (PPD).**

| **Author，year** | **country** | **Type of study** | **Sampling method** | **Timing of postpartum period** | **Maternal characteristics** | **Recruiting background** | **Research site** | **Whether the interviewer is professional** | **Location of the interview** | **Format of the interview** | **Methodology of the interview** | **Diagnostic criteria** | **Sample size** | **major/all depression** | **Quality Score** |
| --- | --- | --- | --- | --- | --- | --- | --- | --- | --- | --- | --- | --- | --- | --- | --- |
| A. A. Leonardou,2009^1^ | Greece | Cohort | Recruitment | 2m | Mixed primiparous and menstrual | Hospital | Urban | Lay interviewers | Non-Hospital | Telephone | SCID | DSM | 81 | 4/10 | 4 |
| Amaury Cantilino,2010^2^ | Brazil | Cross-section | Recruitment | 2-26w | Mixed primiparous and menstrual | Hospital | Urban | Professional | Hospital | Face-to-Face | SCID | DSM | 400 | 29/29 | 5 |
| Annamária Tö reki,2014^3^ | Hungary | Cross-section | Recruitment | 6-8w | Mixed primiparous and menstrual | Hospital | Non-Urban | Lay interviewers | Hospital | Face-to-Face | SCID | DSM | 266 | 8/44 | 4 |
| B. Barnett,1999^4^ | Australia | Cohort | Recruitment | 6w | Mixed primiparous and menstrual | Hospital | Urban | Lay interviewers | Non-Hospital | Face-to-Face | DIS | DSM | 313 | 21/21 | 3 |
| Babu Ram Bhusal,2016^5^ | Nepal | Cross-section | Recruitment | 4-14w | Mixed primiparous and menstrual | Hospital | Urban | Professional | Hospital | Face-to-Face | the diagnostic interview | ICD | 346 | NA/50 | 4 |
| Bonnie WM Siu,2012^6^ | Hong Kong SAR, China | Cohort | Recruitment | 2m | Mixed primiparous and menstrual | Hospital | Urban | Professional | Hospital | Face-to-Face | SCID | DSM | 805 | 126/126 | 4 |
| Carlos Zubaran,2009^7^ | Brazil | Cross-section | Recruitment | 2-12w | Mixed primiparous and menstrual | Hospital | Urban | Lay interviewers | Non-Hospital | Face-to-Face | SCID | DSM | 101 | NA/9 | 3 |
| Catherine Atuhaire,2021^8^ | Uganda | Cross-section | Recruitment | 6-8w | Mixed primiparous and menstrual | Hospital | Non-Urban | Professional | Hospital | Face-to-Face | NA | DSM | 292 | NA/79 | 5 |
| Cemal Akman,2007^9^ | Turkey | Cohort | Recruitment | 6w | Mixed primiparous and menstrual | Hospital | Urban | Professional | Hospital | Face-to-Face | SCID | DSM | 302 | 19/19 | 4 |
| Cheryl Tatano Beck,2001^10^ | The USA | Cross-section | Recruitment | 2-12w | Mixed primiparous and menstrual | Hospital | Urban | Professional | Hospital | Face-to-Face | SCID | DSM | 150 | 18/46 | 3 |
| Cort Pedersen,2016^11^ | The USA | Cohort | Recruitment | 12w | Mixed primiparous and menstrual | Hospital | Urban | Lay interviewers | Non-Hospital | Face-to-Face | MINI | DSM | 199 | 24/24 | 3 |
| Dyanne D. Affonso,1990^12^ | The USA | Cohort | Recruitment | 1-2w | Primiparous | Hospital | Urban | NA | Non-Hospital | Face-to-Face | SADS | RDC | 202 | 2/3 | 3 |
| Ethel Felice,2004^13^ | Malta | Cohort | Random sampling | 8-10w | Mixed primiparous and menstrual | Hospital | Urban | NA | Non-Hospital | Face-to-Face | CIS | ICD | 223 | NA/20 | 5 |
| Gao,2019^14^ | China | Cross-section | Random sampling | 42d | Mixed primiparous and menstrual | Hospital | Urban | Professional | Hospital | Face-to-Face | SCID | DSM | 362 | NA/86 | 3 |
| H. Tissot,2015^15^ | Switzerland | Cross-section | Recruitment | 3m+3w | Mixed primiparous and menstrual | Hospital | Urban | Professional | Non-Hospital | Face-to-Face | DIGS | DSM | 65 | 4/4 | 2 |
| H. Yamashita,2000^16^ | The UK | Cohort | Recruitment | 3w（22-18d） | Mixed primiparous and menstrual | Hospital | Urban | Professional | Non-Hospital | Telephone | SADS | RDC | 88 | 5/12 | 3 |
| Helen Chen,2012^17^ | Singapore | Cross-section | Recruitment | 1-22w | Mixed primiparous and menstrual | Hospital | Urban | Professional | Hospital | Face-to-Face | an unstructured clinical interview | DSM | 487 | 30/30 | 6 |
| J. L. Cox,1982^18^ | The UK | Cohort | Recruitment | 3-5m | Mixed primiparous and menstrual | Hospital | Urban | Professional | Non-Hospital | Face-to-Face | SPI | NA | 103 | 13/13 | 3 |
| J. P.Watson,1984^19^ | The UK | Cohort | Recruitment | 6w | Mixed primiparous and menstrual | Hospital | Urban | Professional | Hospital | Face-to-Face | the standardized psychiatric interview | RDC | 128 | NA/20 | 3 |
| Jane Fisher,2010^20^ | Vietnam | Cross-section | Random sampling | 4-8w | Mixed primiparous and menstrual | Community | Non-Urban | Professional | Non-Hospital | Face-to-Face | SCID | DSM | 165 | NA/24 | 5 |
| K. Yoshida,1997^21^ | The UK | Cohort | Recruitment | Within 3m | Mixed primiparous and menstrual | Community | Urban | Professional | Non-Hospital | Face-to-Face | SADS | RDC | 98 | 6/12 | 3 |
| Karen Wynter,2013^22^ | Australia | Cohort | Recruitment | 6m | Primiparous | Community | Urban | Lay interviewers | Non-Hospital | Telephone | CIDI | DSM | 172 | 1/1 | 3 |
| L. L. Gorman,2004^23^ | Eight high countries | Cross-section | Recruitment | Within 6m | Mixed primiparous and menstrual | Hospital | Urban | Lay interviewers | Hospital | Face-to-Face | SCID | DSM | 261 | 11/21 | 4 |
| Liu,2010^24^ | China | Cross-section | Recruitment | 2-12w | Mixed primiparous and menstrual | Hospital | Urban | NA | NA | NA | SCID | DSM | 387 | 24/111 | 5 |
| M. Agoub,2004^25^ | Morocco | Cohort | Recruitment | 2-3w | Mixed primiparous and menstrual | Hospital | Urban | Professional | Hospital | Face-to-Face | MINI | DSM | 144 | NA/27 | 4 |
| Ma. Asunción Lara,2014^26^ | Mexico | Cohort | Recruitment | 6w | Mixed primiparous and menstrual | Hospital | Urban | Professional | Non-Hospital | Face-to-Face | SCID | DSM | 210 | 29/29 | 2 |
| Mariana Marques,2011^27^ | Portugal | Cohort | Recruitment | 3m | Mixed primiparous and menstrual | Hospital | Urban | NA | Non-Hospital | Face-to-Face | DIGS | DSM | 382 | NA/46 | 4 |
| Meifen Wu,2014^28^ | China | Cohort | Recruitment | Within 3m | NA | Hospital | Urban | NA | Hospital | Face-to-Face | SCID | DSM | 223 | NA/21 | 3 |
| Michael W. O'Hara,1984^29^ | The USA | Cohort | Recruitment | 9w | Mixed primiparous and menstrual | Hospital | Urban | Professional | Hospital | Face-to-Face | SADS | RDC | 98 | 8/12 | 3 |
| Muideen O. Bakare,2014^30^ | Nigeria | Cross-section | Recruitment | 0.03-12m | Mixed primiparous and menstrual | Hospital | Urban | NA | Hospital | Face-to-Face | MINI | ICD | 408 | 62/62 | 3 |
| Nadine Helle,2015^31^ | Germany | Cross-section | Recruitment | 4w | Mixed primiparous and menstrual | Hospital | Urban | Professional | Hospital | Face-to-Face | SCID | DSM | 119 | 3/3 | 4 |
| Nahom Kiros Gebregziabher,2020^32^ | Eritrea | Cross-section | Random sampling | 2-14w | Mixed primiparous and menstrual | Hospital | Non-Urban | Professional | Hospital | Face-to-Face | the MDD interview | DSM | 380 | 28/28 | 6 |
| Nazan Aydin,2004^33^ | Turkey | Cross-section | Recruitment | 1y | Mixed primiparous and menstrual | Hospital | Urban | Professional | Hospital | Face-to-Face | SCID | DSM | 341 | NA/49 | 4 |
| Nigerian,2003^34^ | Nigeria | Cross-section | Recruitment | 6w | Mixed primiparous and menstrual | Hospital | Urban | Professional | Hospital | Face-to-Face | CIDI | ICD | 225 | NA/24 | 4 |
| Pablo Martínez,2016^35^ | Chile | Cohort | Recruitment | 2-6m | Mixed primiparous and menstrual | Hospital | Urban | NA | Non-Hospital | Telephone | MINI | NA | 305 | NA/63 | 4 |
| Paola Benvenuti,1999^36^ | Italy | Cross-section | Random sampling | 8-12w | Mixed primiparous and menstrual | Hospital | Urban | NA | Hospital | Face-to-Face | MINI | DSM | 113 | 6/18 | 2 |
| Patrícia Figueira,2009^37^ | Brazil | Cross-section | Random sampling | 40-90d | Mixed primiparous and menstrual | Hospital | Urban | Lay interviewers | Non-Hospital | Face-to-Face | MINI | DSM | 231 | NA/62 | 5 |
| R. Ghubash,1997^38^ | Arab | Cohort | Recruitment | 8±2w | Mixed primiparous and menstrual | Hospital | Urban | NA | Hospital | Face-to-Face | PSE | NA | 97 | NA/15 | 2 |
| R.J.S. Savarimuthu,2010^39^ | India | Cross-section | Random sampling | 2-10w | Mixed primiparous and menstrual | Community | Non-Urban | Professional | NA | NA | A semi-structured clinical interview | ICD | 137 | NA/36 | 4 |
| R.Kumar,1984^40^ | The UK | Cohort | Recruitment | 3m | Primiparous | Hospital | Urban | Professional | Hospital | Face-to-Face | A semi-structured clinical interview | RDC | 119 | NA/19 | 3 |
| Ricardo Tavares Pinheiro,2013^41^ | Brazil | Cross-section | Random sampling | 45-90d | Mixed primiparous and menstrual | Community | Urban | NA | Non-Hospital | Face-to-Face | MINI | NA | 207 | 27/27 | 5 |
| Sarah Tebeka,2021^42^ | French | Cross-section | Recruitment | 8w; 12m | Mixed primiparous and menstrual | Hospital | Urban | Professional | Non-Hospital | Telephone | DIGS | DSM | 3015 | 250/250 | 6 |
| Stephanie Alves,2018^43^ | Portugal | Cohort | Recruitment | 4m | Mixed primiparous and menstrual | Hospital | Non-Urban | Professional | NA | Telephone | SCID | DSM | 140 | 6/6 | 4 |
| Stephen Matthey,2001^44^ | Australia | Cross-section | Recruitment | 6-7w | Mixed primiparous and menstrual | Hospital | Urban | Lay interviewers | Non-Hospital | Face-to-Face | DIS | DSM | 230 | 11/24 | 4 |
| Susan B. Campbell,1991^45^ | The USA | Cross-section | Recruitment | 6-8w | Primiparous | Hospital | Urban | Professional | Non-Hospital | Telephone | SADS | RDC | 1033 | 66/96 | 4 |
| Susan Pawlby,2008^46^ | The UK | Cohort | Random sampling | 3m | NA | Community | Urban | Lay interviewers | Non-Hospital | Face-to-Face | CIS | ICD | 147 | NA/34 | 3 |
| T. Kitamura,2006^47^ | Japan | Cohort | Recruitment | Within 3m | Primiparous | Hospital | Urban | Lay interviewers | NA | NA | SADS | DSM | 280 | 14/14 | 4 |
| T.J. Leverton,2000^48^ | The UK | Cohort | Recruitment | 3m | Mixed primiparous and menstrual | Hospital | Urban | Professional | Non-Hospital | Face-to-Face | PSE | ICD | 199 | NA/26 | 3 |
| Trang Thu Nguyen,2015^49^ | Vietnam | Cohort | Random sampling | 1y | Mixed primiparous and menstrual | Community | Non-Urban | Professional | Non-Hospital | Face-to-Face | SCID | DSM | 211 | 18/18 | 7 |
| Valerie E,1988^50^ | Canada | Cohort | Recruitment | 6-8w | Mixed primiparous and menstrual | Hospital | Urban | Professional | Non-Hospital | Face-to-Face | SADS | RDC | 115 | 7/19 | 2 |
| Vinit Rahaney,2022^51^ | India | Cross-section | Recruitment | Within 1m | Mixed primiparous and menstrual | Hospital | Non-Urban | NA | Hospital | Face-to-Face | NA | DSM | 60 | 4/4 | 2 |
| Ying Lau,2010^52^ | China | Cross-section | Recruitment | 6-8w | Mixed primiparous and menstrual | Hospital | Urban | Lay interviewers | Hospital | Face-to-Face | SCID | DSM | 342 | NA/16 | 4 |
| Ylva Parfitt,2012^53^ | The UK | Cross-section | Recruitment | 4-8m | Primiparous | Community | Urban | Lay interviewers | Non-Hospital | Face-to-Face | BIMMH | NA | 45 | 5/5 | 3 |
| Ylva Parfitt,2014^54^ | The UK | Cross-section | Recruitment | 5m | Primiparous | Hospital | Urban | Lay interviewers | Non-Hospital | Face-to-Face | BIMMH | NA | 46 | 5/5 | 3 |

Note:

NA=Not available.

BIMMH, The Birmingham Interview for Maternal Mental Health; CIDI, Composite International Diagnostic Interview; CIS, The Clinical Interview Schedule; DIGS, Diagnostic Interview for Genetic Studies; DIS, Diagnostic Inventory Schedule; DSM, Diagnostic and Statistical Manual of Mental Disorders; ICD, International Classification of Diseases; MINI, Mini International Neuropsychiatric Interview; PSE, Present State Examination; RDC, Research Diagnostic Criteria; SADS, Schedule for Affective Disorders and Schizophrenia; SCID, Structured Clinical Interview for DSM; SPI, Standardized Psychiatric Interview.

**STable2：Quality assessment.**

| **Study** | **Are the study design and sampling method appropriate for the research question?** | **Is the sampling frame appropriate?** | **Is the sample size adequate?** | **Are objective, suitable and standard criteria used for measurement of the health outcome?** | **Is the health outcome measured in an unbiased fashion?** | **Is the response rate adequate? Are the refusers described?** | **Are the estimates of prevalence or incidence given with confidence intervals and in detail by subgroup, if appropriate?** | **Are the study subjects and the setting described in detail and similar to those of interest to you?** | **Total scores** |
| --- | --- | --- | --- | --- | --- | --- | --- | --- | --- |
| A. A. Leonardou et al.2009 | 0 | 0 | 0 | 1 | 1 | 1 | 0 | 1 | 4 |
| Amaury Cantilino et al.2010 | 0 | 0 | 1 | 1 | 1 | 0 | 1 | 1 | 5 |
| Annamária Töreki et al.2014 | 0 | 0 | 1 | 1 | 1 | 0 | 0 | 1 | 4 |
| B. Barnett et al.1999 | 0 | 0 | 1 | 1 | 1 | 0 | 0 | 0 | 3 |
| Babu Ram Bhusal,2016 | 0 | 0 | 1 | 1 | 1 | 0 | 0 | 1 | 4 |
| Bonnie WM Siu et al.2012 | 0 | 0 | 1 | 1 | 0 | 1 | 0 | 1 | 4 |
| Carlos Zubaran et al.2009 | 0 | 0 | 0 | 1 | 1 | 0 | 0 | 1 | 3 |
| Catherine Atuhaire,2021 | 0 | 0 | 1 | 0 | 1 | 1 | 1 | 1 | 5 |
| Cemal Akman et al.2007 | 0 | 0 | 1 | 1 | 1 | 0 | 0 | 1 | 4 |
| Cheryl Tatano Beck et al.2001 | 0 | 0 | 0 | 1 | 1 | 0 | 0 | 1 | 3 |
| Cort Pedersen et al.2016 | 0 | 0 | 0 | 1 | 1 | 0 | 0 | 1 | 3 |
| Dyanne D. Affonso et al.1990 | 0 | 0 | 1 | 1 | 0 | 0 | 0 | 1 | 3 |
| Ethel Felice et al.2004 | 1 | 0 | 1 | 1 | 0 | 0 | 1 | 1 | 5 |
| Gao et al.2019 | 0 | 0 | 1 | 1 | 1 | 0 | 0 | 0 | 3 |
| 1. Tissot et al.2015 | 0 | 0 | 0 | 1 | 1 | 0 | 0 | 0 | 2 |
| H. Yamashita et al.2000 | 0 | 0 | 0 | 1 | 1 | 0 | 0 | 1 | 3 |
| Helen Chen et al.2012 | 0 | 0 | 1 | 1 | 1 | 1 | 1 | 1 | 6 |
| J L Cox et al.1982 | 0 | 0 | 0 | 1 | 1 | 0 | 0 | 1 | 3 |
| J. P.Watson et al.1984 | 0 | 0 | 0 | 1 | 1 | 0 | 0 | 1 | 3 |
| Jane Fisher et al.2010 | 0 | 0 | 1 | 1 | 1 | 0 | 1 | 1 | 5 |
| K. Yoshida et al.1997 | 0 | 0 | 0 | 1 | 1 | 0 | 0 | 1 | 3 |
| Karen Wynter et al.2013 | 0 | 0 | 0 | 1 | 1 | 0 | 0 | 1 | 3 |
| L. L. Gorman,2004 | 0 | 0 | 1 | 1 | 1 | 0 | 0 | 1 | 4 |
| Lezhi Li,2011 | 1 | 1 | 1 | 1 | 0 | 0 | 0 | 1 | 5 |
| M. Agoub et al.2004 | 0 | 0 | 0 | 1 | 1 | 1 | 0 | 1 | 4 |
| Ma. Asunción Lara et al.2014 | 0 | 0 | 0 | 1 | 0 | 0 | 0 | 1 | 2 |
| Mariana Marques et al.2011 | 0 | 0 | 1 | 1 | 0 | 0 | 1 | 1 | 4 |
| Meifen Wu et al.2014 | 0 | 0 | 1 | 1 | 0 | 0 | 0 | 1 | 3 |
| Michael W. O'Hara et al.1984 | 0 | 0 | 0 | 1 | 1 | 0 | 0 | 1 | 3 |
| Muideen O. Bakare et al.2014 | 0 | 0 | 1 | 1 | 0 | 0 | 0 | 1 | 3 |
| Nadine Helle et al.2015 | 0 | 0 | 1 | 1 | 1 | 0 | 0 | 1 | 4 |
| Nahom Kiros Gebregziabher et al.2020 | 1 | 1 | 1 | 1 | 1 | 0 | 0 | 1 | 6 |
| Nazan Aydin et al.2004 | 0 | 0 | 1 | 1 | 1 | 0 | 0 | 1 | 4 |
| Nigerian et al.2003 | 0 | 0 | 1 | 1 | 1 | 0 | 0 | 1 | 4 |
| Pablo Martínez et al.2016 | 0 | 1 | 1 | 1 | 0 | 0 | 0 | 1 | 4 |
| Paola Benvenuti et al.1999 | 0 | 0 | 0 | 1 | 0 | 0 | 0 | 1 | 2 |
| Patrícia Figueira et al.2009 | 0 | 0 | 1 | 1 | 1 | 1 | 0 | 1 | 5 |
| R. Ghubash et al.1997 | 0 | 0 | 0 | 1 | 0 | 0 | 0 | 1 | 2 |
| R.J.S. Savarimuthu et al.2010 | 1 | 1 | 0 | 1 | 0 | 0 | 0 | 1 | 4 |
| R Kumar et al.1984 | 0 | 0 | 0 | 1 | 1 | 0 | 0 | 1 | 3 |
| Ricardo Tavares Pinheiro et al.2013 | 1 | 0 | 1 | 1 | 0 | 0 | 1 | 1 | 5 |
| Sarah Tebeka,2021 | 0 | 0 | 1 | 1 | 1 | 1 | 1 | 1 | 6 |
| Stephanie Alves et al.2018 | 0 | 0 | 1 | 1 | 0 | 1 | 0 | 1 | 4 |
| Stephen Matthey et al.2001 | 0 | 0 | 1 | 1 | 1 | 0 | 0 | 1 | 4 |
| Susan B. Campbell and Jeffrey F. Cohn 1991 | 0 | 0 | 1 | 1 | 1 | 0 | 0 | 1 | 4 |
| Susan Pawlby et al.2008 | 1 | 0 | 0 | 1 | 1 | 0 | 0 | 0 | 3 |
| T. Kitamura et al.2006 | 0 | 0 | 1 | 1 | 1 | 0 | 0 | 1 | 4 |
| T.J. Leverton et al.2000 | 0 | 0 | 1 | 1 | 1 | 0 | 0 | 0 | 3 |
| Trang Thu Nguyen et al.2015 | 1 | 1 | 1 | 1 | 1 | 1 | 0 | 1 | 7 |
| Valerie E. Whifien et al.1988 | 0 | 0 | 0 | 1 | 1 | 0 | 0 | 0 | 2 |
| Vinit Rahaney,2022 | 0 | 0 | 1 | 0 | 0 | 0 | 0 | 1 | 2 |
| Ying Lau et al.2010 | 0 | 0 | 1 | 1 | 0 | 1 | 0 | 1 | 4 |
| Ylva Partiff et al.2012 | 0 | 0 | 0 | 1 | 1 | 0 | 0 | 1 | 3 |
| Ylva Parfitt et al. 2014 | 0 | 0 | 0 | 1 | 1 | 0 | 0 | 1 | 3 |

**Reference：**

1. Leonardou AA, Zervas YM, Papageorgiou CC, et al. Validation of the Edinburgh Postnatal Depression Scale and prevalence of postnatal depression at two months postpartum in a sample of Greek mothers. *Journal of Reproductive and Infant Psychology* 2009;27:28–39. doi:10.1080/02646830802004909
2. Cantilino A, Zambaldi CF, Albuquerque TLC de, et al. Postpartum depression in Recife - Brazil: prevalence and association with bio-socio-demographic factors. *J bras psiquiatr* 2010;59:1–9. doi:10.1590/S0047-20852010000100001
3. To¨reki A, Andó B, Dudas RB, et al. Validation of the Edinburgh Postnatal Depression Scale as a screening tool for postpartum depression in a clinical sample in Hungary. *Midwifery* 2014;30:911–8. doi:10.1016/j.midw.2014.02.008
4. Barnett B, Matthey S, Gyaneshwar R. Screening for postnatal depression in women of non-English speaking background. *Archives of Women’s Mental Health* 1999;2:67–74. doi:10.1007/s007370050038
5. Bhusal BR, Bhandari N, Chapagai M, Gavidia T. Validating the Edinburgh Postnatal Depression Scale as a screening tool for postpartum depression in Kathmandu, Nepal. *Int J Ment Health Syst* 2016;10:71. doi:10.1186/s13033-016-0102-6
6. Siu BW, Leung SS, Ip P, Hung SF, O’Hara MW. Antenatal risk factors for postnatal depression: a prospective study of chinese women at maternal and child health centres. *BMC Psychiatry* 2012;12:22. doi:10.1186/1471-244X-12-22
7. Zubaran C, Foresti K, Schumacher MV, et al. Validation of a screening instrument for postpartum depression in Southern Brazil. *Journal of Psychosomatic Obstetrics & Gynecology* 2009;30:244–54. doi:10.3109/01674820903254724
8. Atuhaire C, Rukundo GZ, Nambozi G, et al. Prevalence of postpartum depression and associated factors among women in Mbarara and Rwampara districts of south-western Uganda. *BMC Pregnancy Childbirth* 2021;21:503. doi:10.1186/s12884-021-03967-3
9. Akman C, Uguz F, Kaya N. Postpartum-onset major depression is associated with personality disorders. *Comprehensive Psychiatry* 2007;48:343–7. doi:10.1016/j.comppsych.2007.03.005
10. Beck CT, Gable RK. Further Validation of the Postpartum Depression Screening Scale: *Nursing Research* 2001;50:155–64. doi:10.1097/00006199-200105000-00005
11. Pedersen C, Leserman J, Garcia N, Stansburya M, Meltzer-Brodya S, Johnson J. Late pregnancy thyroid-binding globulin predicts perinatal depression. *Psychoneuroendocrinology* 2016;65:84–93. doi:10.1016/j.psyneuen.2015.12.010
12. Affonso DD, Lovett S, Paul SM, Sheptak S. A Standardized Interview That Differentiates Pregnancy and Postpartum Symptoms from Perinatal Clinical Depression. *Birth* 1990;17:121–30. doi:10.1111/j.1523-536X.1990.tb00716.x
13. Felice E, Saliba J, Grech V, Cox J. Prevalence rates and psychosocial characteristics associated with depression in pregnancy and postpartum in Maltese women. *Journal of Affective Disorders* 2004;82:297–301. doi:10.1016/j.jad.2003.11.011
14. Ming G, Zhi-hui W, Xiao-hong F. Application value of the 9-question depression scale from the patient health questionnaire in the screening of postpartum depression. *CHINA MODERN MEDICINE* 2019;26.
15. Tissot H, Favez N, Frascarolo-Moutinot F, Despland JN. Assessing postpartum depression: Evidences for the need of multiple methods. *European Review of Applied Psychology* 2015;65:61–6. doi:10.1016/j.erap.2015.02.002
16. Yamashita H. Postnatal depression in Japanese women Detecting the early onset of postnatal depression by closely monitoring the postpartum mood. *Journal of Affective Disorders* 2000;58:145–54. doi:10.1016/S0165-0327(99)00108-1
17. Chen H, Bautista D, Ch’ng YC, Li W, Chan E, Rush AJ. Screening for postnatal depression in Chinese-speaking women using the Hong Kong translated version of the Edinburgh Postnatal Depression Scale: Screening for postnatal depression in Asians. *Asia-Pacific Psychiatry* 2013;5:E64–72. doi:10.1111/appy.12080
18. Cox JL, Connor Y, Kendell RE. Prospective Study of the Psychiatric Disorders of Childbirth. *Br J Psychiatry* 1982;140:111–7. doi:10.1192/bjp.140.2.111
19. Watson JP, Elliott SA, Rugg AJ, BROUGH DI. Psychiatric Disorder in Pregnancy and the First Postnatal Year. *Br J Psychiatry* 1984;144:453–62. doi:10.1192/bjp.144.5.453
20. Fisher J, Tran T, La B thi, Kriitmaa K, Rosenthala D, Tran T. Common perinatal mental disorders in northern Viet Nam: community prevalence and health care use. *Bull World Health Organ* 2010;88:737–45. doi:10.2471/BLT.09.067066
21. Yoshida K, Marks MN, Kibe N, Kumar R, Nakano H, Tashiro N. Postnatal depression in Japanese women who have given birth in England. *Journal of Affective Disorders* 1997;43:69–77. doi:10.1016/S0165-0327(96)01419-X
22. Wynter K, Rowe H, Fisher J. Common mental disorders in women and men in the first six months after the birth of their first infant: A community study in Victoria, Australia. *Journal of Affective Disorders* 2013;151:980–5. doi:10.1016/j.jad.2013.08.021
23. Gorman LL, O’Hara MW, Figueiredo B, et al. Adaptation of the Structured Clinical Interview for DSM-IV Disorders for assessing depression in women during pregnancy and post-partum across countries and cultures. *Br J Psychiatry* 2004;184:s17–23. doi:10.1192/bjp.184.46.s17
24. Fang L. Translation and Validation of the Chinese Version of the Postpartum Depression Screening Scale in mothers of Changsha. *Central South University Published Online First*: 2010. doi:10.7666/d.y1721384
25. Agoub M, Moussaoui D, Battas O. Prevalence of postpartum depression in a Moroccan sample. *Arch Womens Ment Health* 2005;8:37–43. doi:10.1007/s00737-005-0069-9
26. Lara MA, Navarrete L, Nieto L, Martín JPB, Navarro JL, Lara-Tapia H. Prevalence and incidence of perinatal depression and depressive symptoms among Mexican women. *Journal of Affective Disorders* 2015;175:18–24. doi:10.1016/j.jad.2014.12.035
27. Marques M, Bos S, Soares MJ, et al. Is insomnia in late pregnancy a risk factor for postpartum depression/depressive symptomatology? *Psychiatry Research* 2011;186:272–80. doi:10.1016/j.psychres.2010.06.029
28. Wu W. Poor Sleep Quality of Third-Trimester Pregnancy is a Risk Factor for Postpartum Depression. *Med Sci Monit* 2014;20:2740–5. doi:10.12659/MSM.891222
29. O'Hara M W , Neunaber D J , Zekoski E M . Prospective study of postpartum depression: prevalence, course, and predictive factors.[J]. *J Abnorm Psychol*, 1984, 93(2):158-171.
30. Bakare MO, Okoye JO, Obindo JT. Introducing depression and developmental screenings into the National Programme on Immunization (NPI) in southeast Nigeria: an experimental cross-sectional assessment. *General Hospital Psychiatry* 2014;36:105–12. doi:10.1016/j.genhosppsych.2013.09.005
31. Helle N, Barkmann C, Bartz-Seel J, et al. Very low birth-weight as a risk factor for postpartum depression four to six weeks postbirth in mothers and fathers: Cross-sectional results from a controlled multicentre cohort study. *Journal of Affective Disorders* 2015;180:154–61. doi:10.1016/j.jad.2015.04.001
32. Gebregziabher NK, Netsereab TB, Fessaha YG, Alaza FA, Ghebrehiwet NK, Sium AH. Prevalence and associated factors of postpartum depression among postpartum mothers in central region, Eritrea: a health facility based survey. *BMC Public Health* 2020;20:1614. doi:10.1186/s12889-02 0-09676-4
33. Aydin N, Inandi T, Yigit A, Hodoglugil NNS. Validation of the Turkish version of the Edinburgh Postnatal Depression Scale among women within their first postpartum year. *Soc Psychiatry Psychiatr Epidemiol* 2004;39. doi:10.1007/s00127-004-0770-4
34. Uwakwe R. Affective (depressive) morbidity in puerperal Nigerian women: validation of the Edinburgh postnatal depression scale: Affective morbidity in puerperal Nigerian women. *Acta Psychiatrica Scandinavica* 2003;107:251–9. doi:10.1034/j.1600-0447.200 3.02477.x
35. Martínez P, Vöhringer PA, Rojas G. Barriers to access to treatment for mothers with postpartum depression in primary health care centers: a predictive model. *Rev Lat Am Enfermagem* 2016;24:e2675. doi:10.1590/1518-8345.0982.2675
36. Benvenuti P. The Edinburgh Postnatal Depression Scale: validation for an Italian sample. *Journal of Affective Disorders* 1999;53:137–41. doi:10.1016/S0165-0327(98)00102-5
37. Patrícia Figueira, Humberto Corrêa, Leandro Malloy-Diniz, Marco Aurélio Romano-Silva. Edinburgh Postnatal Depression Scale for screening in the public health system. *Revista de Saude Publica* 2009;43:Suppl 1:79-84.
38. Ghubash R, Abou-Saleh MT. Postpartum psychiatric illness in Arab culture: Prevalence and psychosocial correlates. *Br J Psychiatry* 1997;171:65–8. doi:10.1192/bjp.171.1.65
39. Savarimuthu RJS, Ezhilarasu P, Charles H, ANTONISAMY B, KURIAN S, JACOB KS. Post-Partum Depression in the Community: a Qualitative Study From Rural South India. *Int J Soc Psychiatry* 2010;56:94–102. doi:10.1177/0020764008097756
40. Kumar R, Robson KM. A Prospective Study of Emotional Disorders in Childbearing Women. *Br J Psychiatry* 1984;144:35–47. doi:10.1192/bjp.144.1.35
41. Tavares Pinheiro R, Monteiro da Cunha Coelho F, Azevedo da Silva R, et al. Association of a serotonin transporter gene polymorphism (5-HTTLPR) and stressful life events with postpartum depressive symptoms: a population-based study. *Journal of Psychosomatic Obstetrics & Gynecology* 2013;34:29–33. doi:10.3109/0167482X.2012.7 59555
42. Tebeka S, Strat YL, Higgons ADP, et al. Prevalence and incidence of postpartum depression and environmental factors: The IGEDEPP cohort. *JOURNAL OF PSYCHIATRIC RESEARCH* 2021;138:366–74. doi:10.1016/j.jpsychires.2021.04.004
43. Alves S, Fonseca A, Canavarro MC, Pereira M. Predictive validity of the Postpartum Depression Predictors Inventory-Revised (PDPI-R): A longitudinal study with Portuguese women. *Midwifery* 2019;69:113–20. doi:10.1016/j.midw.2018.11.006
44. Matthey S, Barnett B, Kavanagh DJ, Howie P. Validation of the Edinburgh Postnatal Depression Scale for men, and comparison of item endorsement with their partners. *Journal of Affective Disorders* 2001;64:175–84. doi:10.1016/S0165-0327(00)00236-6
45. Campbell S B , Cohn J F . Prevalence and correlates of postpartum depression in first-time mothers.[J]. *Journal of Abnormal Psychology*, 1991, 100(4):594-599.
46. Pawlby S, Sharp D, Hay D, O'Keane V. Postnatal depression and child outcome at 11 years: The importance of accurate diagnosis. *Journal of Affective Disorders* 2008;107:241–5. doi:10.1016/j.jad.2007.08.002
47. Kitamura T, Yoshida K, Okano T, et al. Multicentre prospective study of perinatal depression in Japan: incidence and correlates of antenatal and postnatal depression. *Arch Womens Ment Health* 2006;9:121–30. doi:10.1007/s00737-006-0122-3
48. Leverton TJ, Elliott SA. Is the EPDS a magic wand?: 1. A comparison of the Edinburgh Postnatal Depression Scale and health visitor report as predictors of diagnosis on the Present State Examination. *Journal of Reproductive and Infant Psychology* 2000;18:279–96. doi:10.1080/713683048
49. Nguyen TT, Tran TD, Tran T, La B, Nguyen H, Fisher J. Postpartum change in common mental disorders among rural Vietnamese women: Incidence, recovery and risk and protective factors. *Br J Psychiatry* 2015;206:110–5. doi:10.1192/bjp.bp.114.149138
50. Whiffen V E . Vulnerability to postpartum depression: A prospective multivariate study[J]. *Journal of Abnormal Psychology*, 1988, 97(4):467-474.
51. Rahaney V, Faye A, Tadke R, et al. Postpartum depression and its risk factors: A cross-sectional exploratory study. *Ann Indian Psychiatry* 2021;5:36. doi:10.4103/aip.aip_3_21
52. Lau Y, Wang Y, Yin L, Chan KS, Guo X. Validation of the Mainland Chinese version of the Edinburgh Postnatal Depression Scale in Chengdu mothers. *International Journal of Nursing Studies* 2010;47:1139–51. doi:10.1016/j.ijnurstu.2010.02.005
53. Parfitt Y, Ayers S. Postnatal mental health and parenting: The importance of parental anger. *Infant Ment Health J* 2012;33:400–10. doi:10.1002/imhj.21318
54. Parfitt Y, Ayers S. TRANSITION TO PARENTHOOD AND MENTAL HEALTH IN FIRST-TIME PARENTS: Maternal Health and Transition to Parenthood. *Infant Ment Health J* 2014;35:263–73. doi:10.1002/imhj.21443
